# PET117 Deficiency Confers Ferroptosis Resistance Through ACSF2 Downregulation in Cervical Cancer

**DOI:** 10.3390/antiox15070876

**Published:** 2026-07-14

**Authors:** Qiong Sun, Dandan Wang, Qing Zhao, Yu Cui, Yiru Zhang, Yaolu Pi, Huadong Liu, Zhen Wang

**Affiliations:** 1 Shaanxi Belt and Road Joint Laboratory on Gut Microbiome and Cancer, Yulin Hospital, First Affiliated Hospital of Xi’an Jiao Tong University, Yulin 719000, China; qiong_sun@outlook.com; 2 Department of Anesthesiology, Yulin Hospital, First Affiliated Hospital of Xi’an Jiao Tong University, Yulin 719000, China; wangdandan2025@163.com; 3 Shandong Engineering Research Center of Precision Intervention for Aging, Shandong Key Laboratory of Neurorehabilitation, School of Life Sciences and Health, University of Health and Rehabilitation Sciences, Qingdao 266113, China; zhaoqing19990728@163.com (Q.Z.); cy15963006088@163.com (Y.C.); zhangyiru1@uhrs.edu.cn (Y.Z.); pyl0516@hotmail.com (Y.P.)

**Keywords:** PET117, ferroptosis, cervical cancer, mitochondria, ACSF2

## Abstract

Ferroptosis, an iron-dependent form of regulated cell death driven by lipid peroxidation, has emerged as a promising therapeutic strategy for cervical cancer. However, the mitochondrial factors governing ferroptosis sensitivity in this malignancy remain incompletely understood. PET117, a conserved mitochondrial protein, has been implicated in mitochondrial homeostasis, yet its role in ferroptosis regulation and cervical cancer pathophysiology is unknown. Here, we report a novel role of PET117 in regulating ferroptosis. PET117 expression was significantly elevated in cervical cancer tissues and loss of *PET117* in HeLa cells markedly suppressed erastin- and RSL3-induced ferroptosis. Mechanistically, *PET117* deficiency attenuated intracellular reactive oxygen species (ROS) accumulation, lipid peroxidation, and iron overload. Mitochondrial proteomics and RNA-seq revealed extensive remodeling of the mitochondrial proteome and ferroptosis-related transcriptional networks upon PET117 depletion. Notably, integrative analysis of mitochondrial and nascent proteomes identified acyl-CoA synthetase family member 2 (ACSF2) as a downstream target of PET117. These findings establish PET117 as a novel regulator of ferroptosis in cervical cancer, thereby linking mitochondrial function to ferroptosis regulation.

## 1. Introduction

Cervical cancer remains one of the most common gynecological malignancies worldwide and continues to represent a major threat to women’s health despite advances in screening and treatment [1]. Increasing evidence indicates that metabolic reprogramming and oxidative stress play critical roles in cervical cancer progression and therapeutic response [2,3]. Ferroptosis is a distinct form of regulated cell death characterized by iron-dependent lipid peroxidation and has emerged as a critical determinant of cell fate in cancer, neurodegeneration, and metabolic diseases [4,5]. Unlike apoptosis or necroptosis, ferroptosis is tightly linked to cellular redox balance, iron homeostasis, and lipid metabolism, and is regulated by a complex network of metabolic and signaling pathways [6]. Although key regulators such as system Xc^−^ [7], glutathione peroxidase 4 (GPX4) [8], and iron-handling proteins [9] have been extensively characterized, the upstream mechanisms that integrate mitochondrial function with ferroptotic signaling remain incompletely understood [10,11].

Mitochondria have emerged as critical organelles in ferroptosis regulation, contributing to iron homeostasis, lipid metabolism, and ROS generation [12,13]. However, the repertoire of mitochondrial proteins modulating ferroptosis sensitivity remains largely undefined [14,15]. Identifying mitochondrial regulators that link mitochondrial homeostasis to ferroptosis cell death is therefore essential for understanding how ferroptosis is controlled under physiological and pathological conditions. PET117 is a mitochondrial protein required for the assembly and maturation of cytochrome c oxidase (complex IV), thereby maintaining efficient mitochondrial oxidative phosphorylation and respiratory chain activity [16]. Loss of PET117 impairs complex IV assembly, resulting in altered mitochondrial respiration and disruption of cellular redox homeostasis [17]. Increasing evidence indicates that mitochondrial metabolism critically determines ferroptosis susceptibility through modulation of electron transport chain activity, mitochondrial ROS production, iron metabolism, and lipid peroxidation [12,18,19]. Given the indispensable role of PET117 in maintaining mitochondrial respiratory function, disruption of PET117 may alter mitochondrial redox balance and consequently influence cellular sensitivity to ferroptosis. However, whether PET117 participates in ferroptosis regulation and the underlying molecular mechanisms remain unknown.

In this study, we uncover an unexpected role of PET117 in promoting ferroptosis in cervical cancer. Through integrated proteomic, transcriptomic, and functional analyses, we demonstrate that PET117 deficiency confers resistance to ferroptosis inducers and identify ACSF2 as a potential downstream effector. These findings establish PET117 as a previously unrecognized component of the ferroptosis regulatory network and expand the functional landscape of this mitochondrial protein.

## 2. Materials and Methods

### 2.1. Antibodies and Reagents

The following primary antibodies were used in this study: PET117 (Invitrogen, PA5-61574, Carlsbad, CA, USA), β-ACTIN (Cell Signaling Technology, #3700S, Danvers, MA, USA), α-TUBULIN (Cell Signaling Technology, #3873S, Danvers, MA, USA), SDHB (Santa cruz, #271548, Santa Cruz, CA, USA), Histone H3 (Abcam, #ab1791, Cambridge, UK), Caspase3 (Cell Signaling Technology, #14220S, Danvers, MA, USA), PARP (Cell Signaling Technology, #9532S, Danvers, MA, USA), BCL-2 (Cell Signaling Technology, #3498S, Danvers, MA, USA), LC3B (Cell Signaling Technology, #3868, Danvers, MA, USA), P62 (Abclonal, #A19700, Wuhan, China), Parkin (Abclonal, #A0968), ACSF2 (Proteintech, # 16140-1-AP, Wuhan, China), and GPX4 (Proteintech, # 30388-1-AP, Wuhan, China). Reagents used in this study include erastin (Millipore, #329600, Burlington, MA, USA), RSL3 (Sigma, #SML2234, St. Louis, MO, USA), ferrostatin-1 (MCE, #HY-100579, Monmouth Junction, NJ, USA), and puromycin (Gibco, #2259656, Grand Island, NY, USA).

### 2.2. Data Acquisition and Bioinformatics Analysis

GSE63514 dataset was retrieved from the GEO database (https://www.ncbi.nlm.nih.gov/geo (accessed on 6 July 2024)). The GSE63514 dataset included 24 normal samples and 28 cervical cancer samples, sequenced using the GPL570 platform. Transcriptome profiles of the TCGA-CESC cohort were downloaded from the TCGA database. The expression matrix was preprocessed and normalized before analysis. Ferroptosis-related genes were obtained from FerrDb ferroptosis database (FerrDb) and previous studies. (http://www.zhounan.org/ferrdb/ (accessed on 6 February 2024)). Spearman correlation analysis was performed to evaluate the association between PET117/ACSF2 and ferroptosis-related genes across all samples using the cor.test() function in R (version 4.3.3).

### 2.3. Cell Culture

The HeLa cells used in this study were purchased from the China Center for Type Culture Collection (CCTCC, Wuhan, China; Accession Number: TCHu187). HeLa cells were cultured in normal H-DMEM (VivaCell, #C3113-0500, Shanghai, China) medium supplemented with 10% (*v*/*v*) fetal bovine serum (VivaCell, #C04001-500) and 1X penicillin–streptomycin (VivaCell, #C0222, Shanghai, China). HeLa cells were incubated at 37 °C under 5% CO_2_ humidified atmosphere (Thermo Scientific, Waltham, MA, USA).

### 2.4. Preparation of PET117-Knockout Cell Line

*PET117*-knockout (*PET117*-KO) HeLa cells were generated using CRISPR/Cas9-mediated genome editing as previously described [20,21,22] and our earlier publication [23]. In those studies, the CRISPR/Cas9-mediated genome editing strategy, sgRNA sequences, sequencing validation, and characterization of PET117 deficiency were comprehensively described. Briefly, guide RNAs (gRNAs) targeting the PET117 gene (forward: 5′-CTGCTGCTTCACATGTACGC-3′; reverse: 5′-CGTACATGTGAAGCAGCAGT-3′) were cloned into the lentiCRISPR v2 vector (Addgene, #52961, Watertown, MA, USA). For lentiviral production, the lentiCRISPR v2 constructs were co-transfected into HEK293T cells together with the packaging plasmids pVSVG and pD8.9 using Polyethylenimine HCl MAX transfection reagent (Polysciences, #24765, Warrington, PA, USA) according to the manufacturer’s instructions. Viral supernatants were collected at 48 h and 72 h post-transfection and filtered through a 0.45 μm membrane filter. HeLa cells at approximately 30% confluence were infected with the collected lentiviral supernatants for 72 h, followed by selection with puromycin (2 μg/mL) for 5–7 days to establish stable cell populations. Single-cell clones were subsequently isolated and expanded. Successful knockout of PET117 was confirmed by Western blot analysis using an anti-PET117 antibody.

### 2.5. Cell Viability Assay

Control and *PET117*-KO HeLa cells were seeded in 96-well plates at a density of 2.5 × 10^3^ per well for 16–18 h. To induce ferroptosis, cells were treated with erastin (40 μM) or RSL3 (4 μM) for 24 h. Subsequently, 100 μL of a mixture containing culture medium and Cell Counting Kit-8 (CCK-8) solution (Beyotime Biotechnology, Shanghai, China) was added to each well. After an additional incubation for 0.5–1 h (37 °C, 5% CO_2_), the absorbance at 450 nm was measured using a microplate reader (Thermo Fisher Scientific, Waltham, MA, USA).

### 2.6. ROS Determination

Control and *PET117*-KO HeLa cells were seeded into 6-well plates at a density of 5 × 10^5^ cells per well. After a 24 h incubation, the cells were treated with RSL3 (4 μM) for 6 h, intracellular ROS levels were determined using a dichlorodihydrofluorescein diacetate (DCFH-DA) probe (#S0033S, Beyotime, Shanghai, China). Briefly, the DCFH-DA probe was diluted 1:1000 in serum-free medium and incubated with the cells at 37 °C for 30 min. Following incubation, the cells were harvested via trypsinization and collected into 1.5 mL microcentrifuge tubes. The fluorescence intensity was subsequently quantified using a NovoCyte flow cytometer (ACEA Biosciences, San Diego, CA, USA).

### 2.7. Intracellular Ferrous Ion (Fe^2+^) Assay

Intracellular ferrous ions (Fe^2+^) were detected using the RhoNox-6 fluorescent probe (#S1070S, Beyotime, Shanghai, China) according to the manufacturer’s protocol. Briefly, Control and *PET117*-KO HeLa cells were seeded into 12-well plates at a density of 3 × 10^4^ cells/well. Following a 24 h incubation, cells were treated with RSL3 (4 μM) for 6 h to induce ferroptosis. Subsequently, the cells were incubated with RhoNox-6 (1:1000 dilution in serum-free medium) at 37 °C for 30 min. After washing with PBS, Fe^2+^ levels were visualized using an inverted fluorescence microscope (Leica Biosystems). The fluorescence intensity was quantified using ImageJ software (version 2.0.0-rc-69/1.52p).

### 2.8. Malondialdehyde (MDA) Assay

MDA levels were quantified using a Lipid Peroxidation MDA Assay Kit (A003-4-1, Nanjing Jiancheng Bioengineering Institute, Nanjing, China) following the manufacturer’s protocol. Briefly, about 1 × 10^6^ Control or *PET117*-KO HeLa were lysed via ultrasonication. The resulting lysates were incubated in a water bath at 95 °C for 40 min. Upon cooling to room temperature, the samples were centrifuged, and the supernatants were harvested. The absorbance was subsequently measured using a microplate reader (Thermo Fisher Scientific, Waltham, MA, USA).

### 2.9. 4-Hydroxynonenal (4-HNE) Assay

Briefly, about 5 × 10^6^ Control or *PET117*-KO HeLa cells were harvested to determine 4-HNE levels using a commercial assay kit (H268-1-2, Nanjing Jiancheng Bioengineering Institute, China) in accordance with the manufacturer’s instructions. The absorbance of the resulting samples was subsequently quantified using a microplate reader (Thermo Fisher Scientific, Waltham, MA, USA).

### 2.10. Quantitative Real-Time PCR

Total RNA was isolated with RNAiso Plus (Thermo Fisher Scientific, #15596026CN, Waltham, MA, USA). Subsequently, 500 ng of RNA was reverse-transcribed into cDNA using the PrimeScript RT Master Mix Kit (TaKaRa, #RR036A, Kusatsu, Shiga, Japan) in a 20 μL system. The resulting cDNA was used for qRT-PCR analysis with the TB Green^®^ Premix Ex Taq™ II Kit (TaKaRa, #RR820A, Kusatsu, Shiga, Japan). Detailed information regarding primer sequences is provided in Supplementary Table S4.

### 2.11. RNA Sequencing (RNA-Seq)

Total RNA was extracted from 1 × 10^6^ Control or *PET117*-KO HeLa cells using 1.5 mL of RNAiso Plus (Thermo Fisher Scientific, #15596026CN, Waltham, MA, USA). The isolated RNA samples were transported on dry ice to Novogene Co., Ltd. (Beijing, China) for high-throughput RNA sequencing services. Following stringent quality control, transcriptome libraries were constructed and subjected to paired-end sequencing on the Illumina HiSeq platform. The resulting clean reads were aligned to the reference genome using HISAT2, and gene expression levels were quantified as Fragments Per Kilobase of transcript per Million mapped reads (FPKM) using HTSeq. Statistical visualization, including the advanced volcano plot, was performed using the OmicStudio platform (https://www.omicstudio.cn/tool/ (accessed on 20 February 2026)).

### 2.12. Mitochondrial Isolation and Proteomic Sample Preparation

About 5 × 10^5^ control or *PET117*-KO HeLa cells were seeded into 6-well plates. After incubation for 24 h, cells were washed with PBS and partitioned into mitochondrial, cytosolic, and nuclear fractions using a commercial cell fractionation kit (Abcam, #ab109819) according to the manufacturer’s protocol. Equal volumes of the mitochondrial fractions were subjected to alkylation, after which protein precipitation was induced by adding five volumes of precipitation buffer (acetone: ethanol: acetic acid = 50:50:0.1, *v*/*v*/*v*). The resulting protein pellets were resuspended in 200 μL of 8 M urea containing 100 mM ammonium bicarbonate (pH 7.5). Protein concentrations were determined via the Bradford assay. Finally, equal amounts of protein were digested with trypsin at a 50:1 (*w*/*w*) ratio for 18 h at 37 °C. The prepared peptides were analyzed and identified using liquid chromatography–tandem mass spectrometry (LC–MS/MS).

### 2.13. Metabolic Labeling of Nascent Proteome

The metabolic labeling of nascent proteins was conducted as previously described [24]. Briefly, approximately 1 × 10^7^ HeLa cells were starved in methionine-free and serum-free DMEM for 2 h to deplete endogenous methionine. Subsequently, cells were pulse-labeled with either 0.4 mM azidohomoalanine (AHA) or methionine (as a control) in DMEM for 4 h. Following incubation, cells were harvested and lysed in a protein extraction buffer (8 M urea, 100 mM NH_4_HCO_3_, 0.1% Triton X-100 and complete protease inhibitor cocktail 1X, pH 7.5). The click reaction was performed overnight with 2 mM alkynylated biotin in click reaction buffer (TBTA: 100 μM, CuSO_4_: 4 mM, TCEP: 2 mM) and after the click reaction, five volumes of precipitation buffer (acetone: alcohol: acetic acid = 50:50:1) were added to the samples and incubated at −20 °C overnight. Non-precipitate was removed by centrifugation at 20,000× *g*, 4 °C for 30 min. The precipitate was subsequently washed with ice-cold acetone and 75% ice-cold ethanol and resuspended in 8 M urea in 100 mM NH_4_HCO_3_. Nascent proteome was enriched by streptavidin magnetic beads (Pierce, #88816, Thermo Fisher Scientific, Waltham, MA, USA) on a rotator at 4 °C, overnight.

### 2.14. Mass Spectra and Protein Identification

Peptides were separated using an UltiMate 3000 RSLCnano system (Thermo Fisher Scientific, Waltham, MA, USA) equipped with a C18 reverse-phase analytical column (750 μm × 150 mm, 3 μm particle size). Mass spectrometric analysis was performed on a Q-Exactive Plus MS (Thermo Fisher Scientific, Waltham, MA, USA) in positive ion mode. The electrospray voltage was maintained at 2.3 kV, and the ion transfer tube temperature was set to 320 °C. The resulting MS data were processed using MaxQuant (v.1.6.0.1). Protein identification was performed against a database retrieved from UniProt (www.uniprot.org/ (accessed on 14 October 2022)).

### 2.15. Statistical Analysis

Statistical analyses were performed using GraphPad 8.0. Data are presented as mean ± standard error of the mean (SEM). Differences between two groups were evaluated using a two-tailed Student’s *t*-test assuming equal variances (T.TEST function, tails = 2, type = 2). For the omics datasets, proteins with a *p* value < 0.05 were considered significantly changed and were selected for downstream analyses. No additional multiple-testing correction was applied because the present omics analysis was exploratory in nature.

## 3. Results

### 3.1. PET117 Is Upregulated in Cervical Cancer and Associated with Ferroptosis-Related Signaling

**Figure 1:**
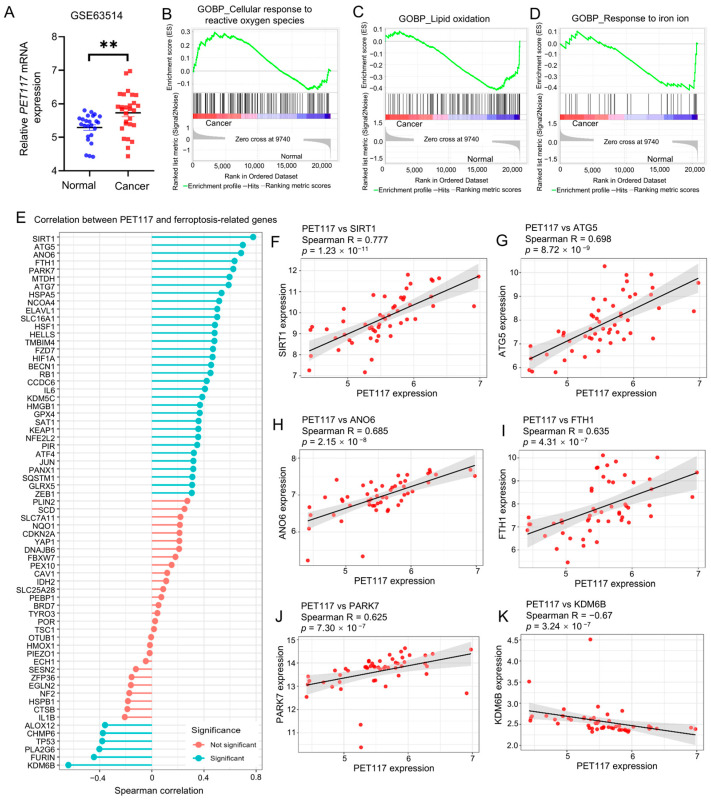
*PET117* is upregulated in cervical cancer and associated with ferroptosis-related signaling. (**A**) Differential expression analysis of *PET117* in normal cervical tissues (*n* = 24) and cervical cancer tissues (*n* = 28) from the GSE63514 dataset. Relative mRNA expression levels are shown, and statistical significance was evaluated using a Student’s *t*-test assuming equal variances. ** *p* < 0.01. (**B**–**D**) Gene set enrichment analysis (GSEA) based on *PET117* expression in GSE63514 samples. (**E**) Spearman correlation analysis between *PET117* and ferroptosis-related genes in cervical cancer samples. Positive and negative correlations are shown, with significantly correlated genes highlighted in cyan and non-significant genes in salmon. (**F**–**K**) Representative scatter plots showing the strongest correlations between PET117 and selected ferroptosis-related genes, including positively correlated genes. Correlation coefficients (Spearman R) and corresponding *p* values are indicated in each panel.

To investigate the potential role of PET117 in cervical cancer, we first analyzed its expression in the GSE63514 dataset obtained from the GEO database. PET117 mRNA expression was significantly upregulated in cervical cancer tissues compared with normal cervical tissues (Figure 1A). Consistent with this finding, analysis of the TCGA-CESC cohort using the GEPIA2 platform further confirmed that PET117 expression was significantly elevated in cervical cancer tissues relative to normal cervical tissues (Supplementary Figure S1A). However, Kaplan–Meier survival analysis demonstrated that PET117 expression was not significantly associated with overall survival in cervical cancer patients (Supplementary Figure S1B), suggesting that PET117 may contribute to cervical cancer progression through its biological functions rather than serving as an independent prognostic biomarker.

To gain mechanistic insight into the functional consequences of PET117 upregulation, we performed gene set enrichment analysis (GSEA) using the GSE63514 dataset. Remarkably, high PET117 expression was significantly enriched in pathways directly implicated in ferroptosis regulation, including cellular response to ROS, lipid oxidation, and response to iron ion (Figure 1B–D). These pathways represent the major biochemical features of ferroptosis, namely oxidative stress, lipid peroxidation, and iron-dependent reactions, suggesting a potential role for PET117 in ferroptosis regulation. We next examined the correlation between PET117 and ferroptosis-related genes using Spearman correlation analysis (Figure 1E), ferroptosis-associated genes were retrieved from the FerrDb database (http://www.zhounan.org/ferrdb/ (accessed on 6 February 2024)) [25]. The genes showing the strongest positive correlations with PET117 included SIRT1, ATG5, ANO6, FTH1 and PARK7, whereas KDM6B exhibited the strongest negative correlation. (Figure 1F–K), which totally suggested a positive role of PET117 in ferroptosis. Collectively, these bioinformatic analyses suggest that PET117 may participate in the regulation of ferroptosis in cervical cancer.

### 3.2. Loss of PET117 Confers Resistance to Erastin- and RSL3-Induced Ferroptosis

**Figure 2:**
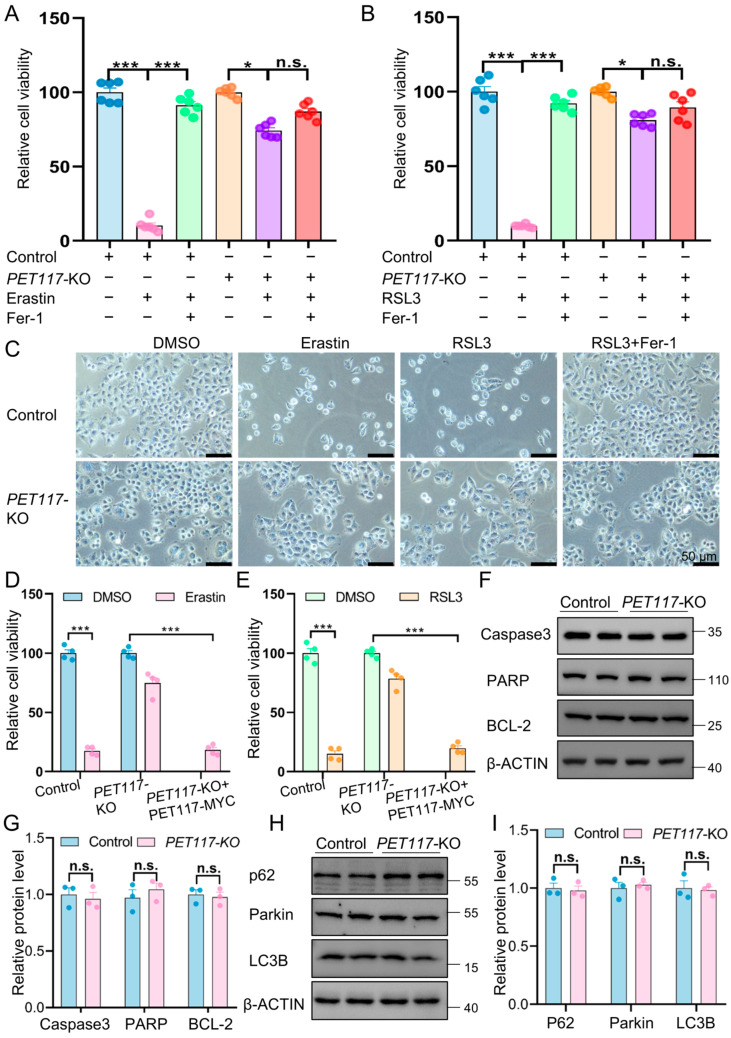
Loss of *PET117* confers resistance to erastin- and RSL3-induced ferroptosis. (**A**) Control and *PET117*-KO HeLa cells were treated with erastin (40 μM), Fer-1 (10 μM) or their combination for 24 h. Cell viability was determined by the CCK-8 assay and analyzed by one-way ANOVA (*n* = 6, *: *p* < 0.05, ***: *p* < 0.001, n.s., not significant). (**B**) Control and *PET117*-KO HeLa cells were treated with RSL3 (4 μM), Fer-1 (10 μM) or their combination for 24 h. Cell viability was determined by the CCK-8 assay (*: *p* < 0.05, ***: *p* < 0.001, n.s., not significant). (**C**) Cell morphology was observed under an inverted light microscope. Scale bar = 50 µm. (**D**,**E**) Relative cell viability of control, *PET117*-KO and *PET117*-KO HeLa cells expressing exogenous PET117 were treated with erastin (**D**) or RSL3 (**E**) (*n* = 4, ***: *p* < 0.001, n.s). (**F**,**G**) Immunoblotting assays of Caspase3, PARP, and BCL-2 in the control and *PET117*-KO HeLa cells. β-ACTIN was used as loading controls. Quantified intensities were presented in bar graph (**G**) as means ± s.e.m. and analyzed by a two-tailed paired Student’s *t*-test (*n* = 3, n.s., not significant). (**H**,**I**) Immunoblotting assays of p62, Parkin, and in the control and *PET117*-KO HeLa cells. β-ACTIN was used as loading controls. Quantified intensities were presented in bar graph (**I**) as means ± s.e.m. and analyzed by a two-tailed paired Student’s *t*-test (*n* = 3, n.s., not significant).

To elucidate the functional role of PET117, we generated *PET117* knockout (KO) HeLa cell lines using the CRISPR/Cas9 system described previously [23] (Supplementary Figure S2). We next investigated whether PET117 influences cellular sensitivity to ferroptosis induced by erastin or RSL3. After 24 h of treatment, control cells exhibited extensive cell death. However, *PET117*-KO cells displayed markedly increased survival under both conditions (Figure 2A–C). Co-treatment with the ferroptosis-specific inhibitor ferrostatin-1 (Fer-1) effectively rescued both control and *PET117*-KO cells from RSL3-induced lethality, confirming the cell death modality as ferroptosis (Figure 2A–C). Crucially, the re-expression of PET117 in the *PET117*-KO cells restored sensitivity to erastin- and RSL3-mediated ferroptosis (Figure 2D,E), confirming that PET117 is essential for the ferroptotic response in this model.

To determine whether *PET117* deficiency affected other forms of cell death, we examined apoptosis- and autophagy-related markers. Immunoblot analysis revealed no significant differences in the expression of Caspase-3, PARP and BCL-2 between control and *PET117*-KO cells (Figure 2F,G). Similarly, analysis of autophagy markers, including p62, Parkin and LC3B, revealed no substantial differences between control and *PET117*-KO cells (Figure 2H,I). These results indicate that PET117 specifically regulates ferroptosis rather than apoptosis or autophagy.

### 3.3. PET117 Deficiency Attenuates ROS Accumulation, Lipid Peroxidation, and Iron Accumulation

**Figure 3:**
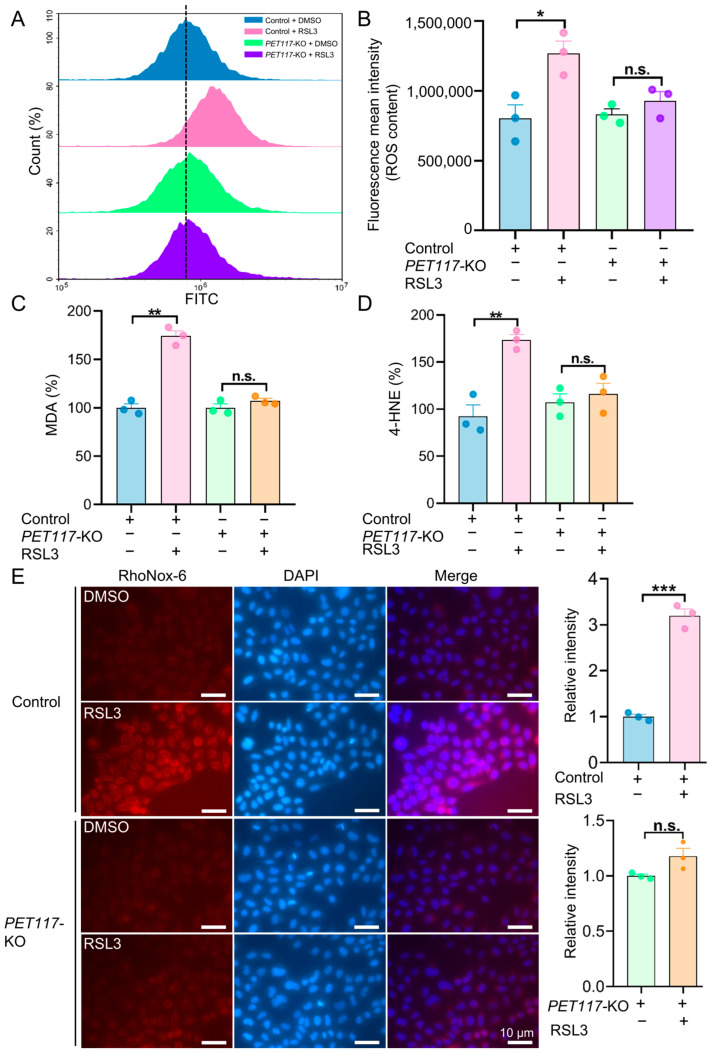
*PET117* deficiency attenuates ROS accumulation, lipid peroxidation, and iron accumulation. (**A**) Control and *PET117*-KO HeLa were treated with RSL3 (4 µM) for 6 h. The cellular ROS level was analyzed by a flow cytometer using DCFH-DA. (**B**) The fluorescence mean intensity was presented in bar graph as means ± s.e.m. and analyzed by a two-tailed paired Student’s *t*-test (*n* = 3, *: *p* < 0.05, n.s., not significant). (**C**) Control and *PET117*-KO HeLa were treated with RSL3 (4 μM) for 6 h. Intracellular MDA levels were presented in bar graph as means ± s.e.m. and analyzed by a two-tailed paired Student’s *t*-test (*n* = 3, **: *p* < 0.01, n.s., not significant). (**D**) Control and *PET117*-KO HeLa were treated with RSL3 (4 μM) for 6 h. Intracellular 4-HNE levels were presented in bar graph as means ± s.e.m. and analyzed by a two-tailed paired Student’s *t*-test (*n* = 3, **: *p* < 0.01, n.s., not significant). (**E**) Control and *PET117*-KO HeLa were treated with RSL3 (4 μM) for 6 h. Chelatable iron levels were determined using RhoNox-6. Scale bar = 10 µm. The relative fluorescence intensity was presented in bar graph as means ± s.e.m. and analyzed by a two-tailed paired Student’s *t*-test (*n* = 3, ***: *p* < 0.001, n.s., not significant). MDA, malondialdehyde; 4-HNE, 4-hydroxynonenal.

Ferroptosis is characterized by excessive ROS production, lipid peroxidation, and intracellular iron accumulation [5,26]. We therefore examined these hallmarks in RSL3-treated cells. While control cells exhibited a robust increase in intracellular ROS levels, this induction was markedly attenuated in *PET117*-KO cells (Figure 3A,B). Consistently, the lipid peroxidation markers malondialdehyde (MDA) [27] and 4-hydroxynonenal (4-HNE) [28] were significantly elevated in control cells following RSL3 exposure but remained largely unchanged in *PET117*-KO cells (Figure 3C,D). Furthermore, the RSL3-induced accumulation of labile iron was pronounced in control cells but significantly blunted in the absence of *PET117* (Figure 3E).

### 3.4. PET117 Deficiency Remodels the Mitochondrial Proteome and Alters Ferroptosis-Related Pathways

**Figure 4:**
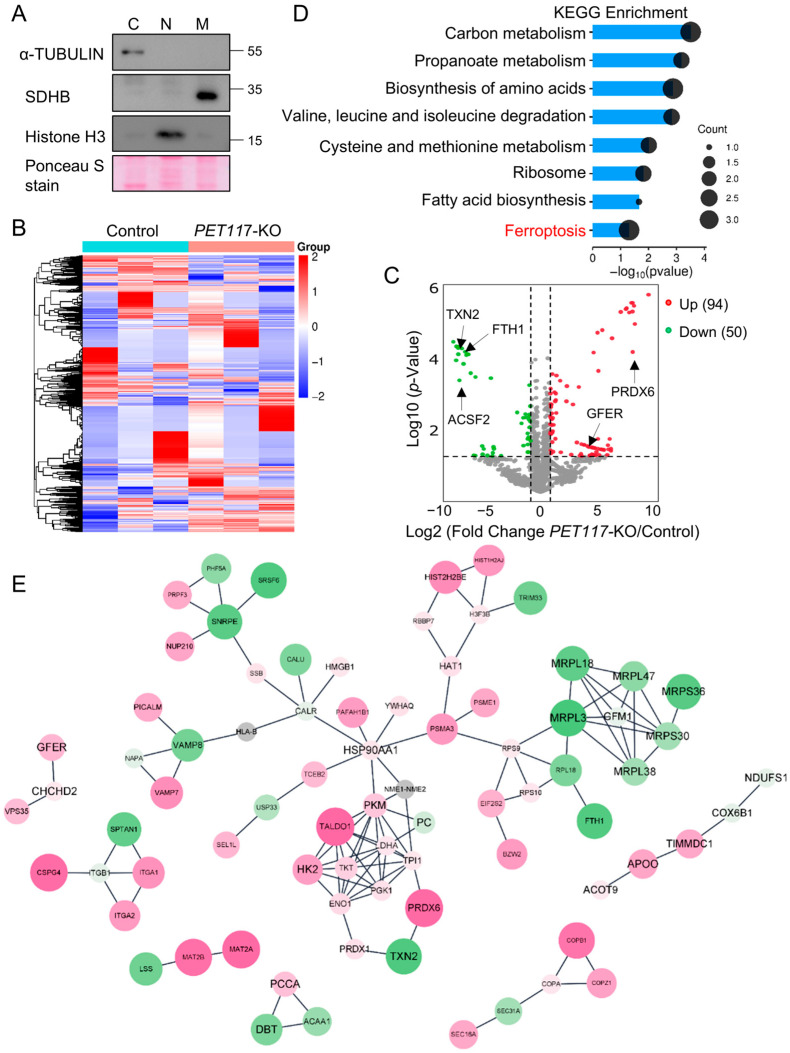
*PET117* deficiency remodels the mitochondrial proteome and alters ferroptosis-related pathways. (**A**) Immunoblotting assays of α-TUBULIN, SDHB, and Histone H3 in subcellular fractionation samples. (**B**) Heatmaps of hierarchical clustering in protein levels of the control and *PET117*-KO HeLa cells (*n* = 3 biologically independent samples). (**C**) Volcano map of the control and *PET117*-KO HeLa cells. Proteins with *p*-value < 0.05 are present above the horizontal lines (gray). Proteins with significant fold-change are outside the gray vertical lines (log2-fold change ≥ 1, *p*-value < 0.05, red; log2 fold change ≤ −1, *p*-value < 0.05, green; *n* = 3 biologically independent samples). (**D**) KEGG enrichment analysis of proteins with significant difference (*PET117*-KO versus control, cut-off at |log2-fold change| ≥ 1 and *p*-value < 0.05). (**E**) PPI network. The red indicates upregulated proteins. The green indicates downregulated proteins. The larger dots represent greater fold changes, and larger font sizes indicate stronger associations of the proteins with mitochondria (*n* = 3 biologically independent samples). C, cytosolic; N, nuclear; M, mitochondria; KEGG, Kyoto Encyclopedia of Genes and Genomes.

As PET117 is a mitochondrial protein, we then investigated whether its loss alters the mitochondrial proteome. Mitochondrial fractions were isolated from control and *PET117*-KO cells for quantitative proteomic analysis. The purity of the isolated mitochondria was confirmed by the enrichment of SDHB and the absence of cytoplasmic (α-TUBULIN) or nuclear (Histone H3) markers (Figure 4A). Quantitative proteomics characterized approximately 2500 proteins (Supplementary Table S1), revealing a profound remodeling of the mitochondrial proteome upon *PET117* depletion (Figure 4B). Differentially expressed proteins (DEPs) were determined via volcano plot analysis, using cut-off criteria of |log_2_ fold change| ≥ 1 and *p*-value < 0.05 (Figure 4C). KEGG pathway enrichment analysis indicated that these DEPs are significantly involved in “amino acid biosynthesis,” “cysteine metabolism,” and “ferroptosis” (Figure 4D). To uncover the functional relationships among these significantly changed proteins, high-confidence interactions were retrieved from the STRING database and visualized via Cytoscape (version 3.10.3) (Figure 4E). Consistent with these enrichment results, *PET117* deficiency induced substantial changes in redox- and ferroptosis-related mitochondrial proteins. Specifically, the mitochondrial thioredoxin 2 (TXN2) was significantly downregulated, whereas the antioxidant enzyme peroxiredoxin 6 (PRDX6) and peroxide-responsive proteins coiled-coil-helix-coiled-coil-helix domain containing 2 (CHCHD2) and growth factor erv1-like (GFER) were upregulated. In parallel, ferritin heavy chain 1 (FTH1) and ACSF2 were markedly decreased in *PET117*-KO mitochondria (Figure 4C). Together, these findings suggest that PET117 regulates ferroptosis through coordinated modulation of mitochondrial redox homeostasis, iron metabolism, and lipid metabolic pathways.

### 3.5. PET117 Deficiency Induces Transcriptional Reprogramming of Ferroptosis-Related Genes

**Figure 5:**
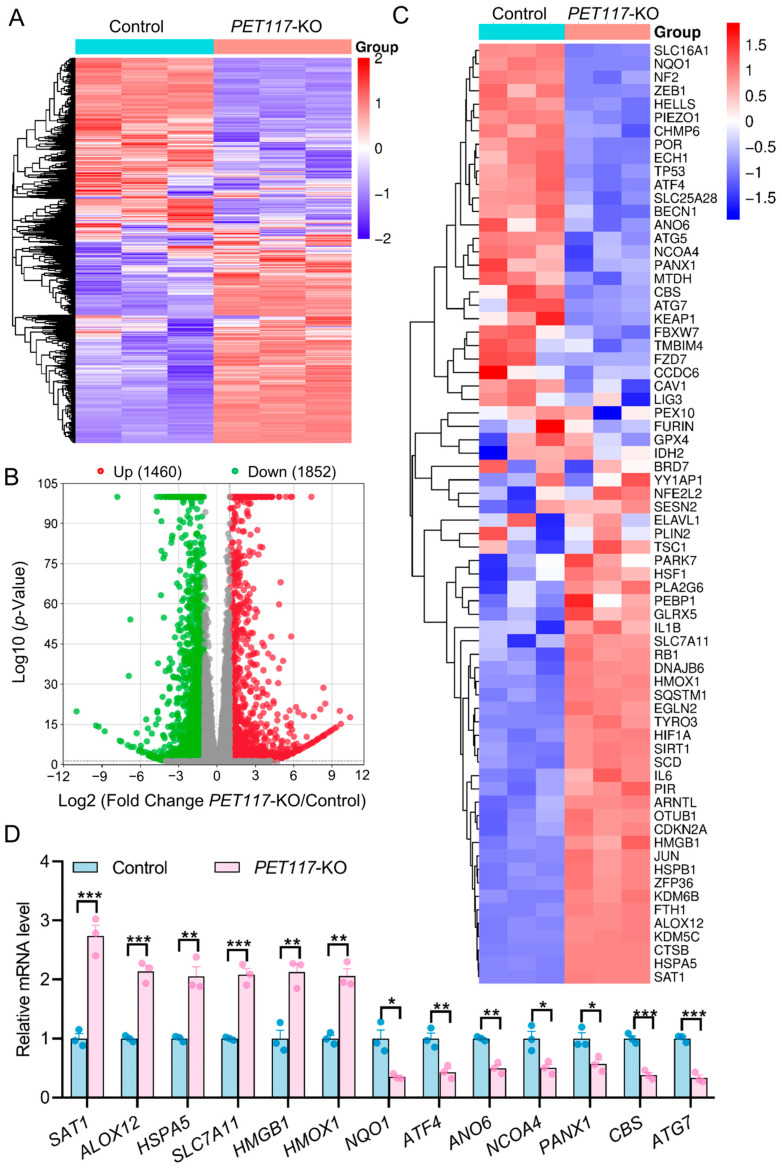
*PET117* deficiency induces transcriptional reprogramming of ferroptosis-related genes. (**A**) Heatmaps of hierarchical clustering in gene levels of the control and *PET117*-KO HeLa cells (*n* = 3 biologically independent samples). (**B**) Volcano map of the control and *PET117*-KO HeLa cells. Genes with *p*-value < 0.05 are present above the horizontal lines (gray). Genes with significant fold-change are outside the gray vertical lines (log2 fold change ≥ 1, *p*-value < 0.05, red; log2 fold change ≤ −1, *p*-value < 0.05, green; *n* = 3 biologically independent samples). (**C**) Heatmap of the expression levels of ferroptosis-associated genes from two groups based on RNA-seq. (**D**) qRT-PCR assays of SAT1, ALOX12, HSPA5, CTSB, HMGB1, HMOX1, NQO1, ATF4, ANO6, NCOA4, PANX1, CBS and SLC7A11 mRNAs in control and *PET117*-KO HeLa cells (*n* = 3, *: *p* < 0.05, **: *p* < 0.01, ***: *p* < 0.001).

To investigate whether PET117 deficiency affects the transcriptional landscape of ferroptosis-related genes, we performed RNA sequencing (RNA-seq) analysis. Approximately 15,000 protein-coding genes were identified (Supplementary Table S2), and heatmap analysis revealed extensive transcriptional alterations following PET117 depletion (Figure 5A). Differentially expressed genes were determined via volcano plot analysis, using cut-off criteria of |log_2_ fold change| ≥ 1 and *p*-value < 0.05 (Figure 5B). To specifically evaluate ferroptosis-related transcriptional changes, ferroptosis-associated genes were retrieved from the FerrDb database. Examination of their expression profiles in the RNA-seq dataset demonstrated that *PET117* deficiency markedly altered the transcriptional abundance of multiple ferroptosis-related genes (Figure 5C). These transcriptional changes were further validated by quantitative real-time PCR (qPCR), and the results were consistent with the RNA-seq data (Figure 5D).

### 3.6. PET117 Promotes Ferroptosis Partially Through Maintaining ACSF2 Protein Abundance

**Figure 6:**
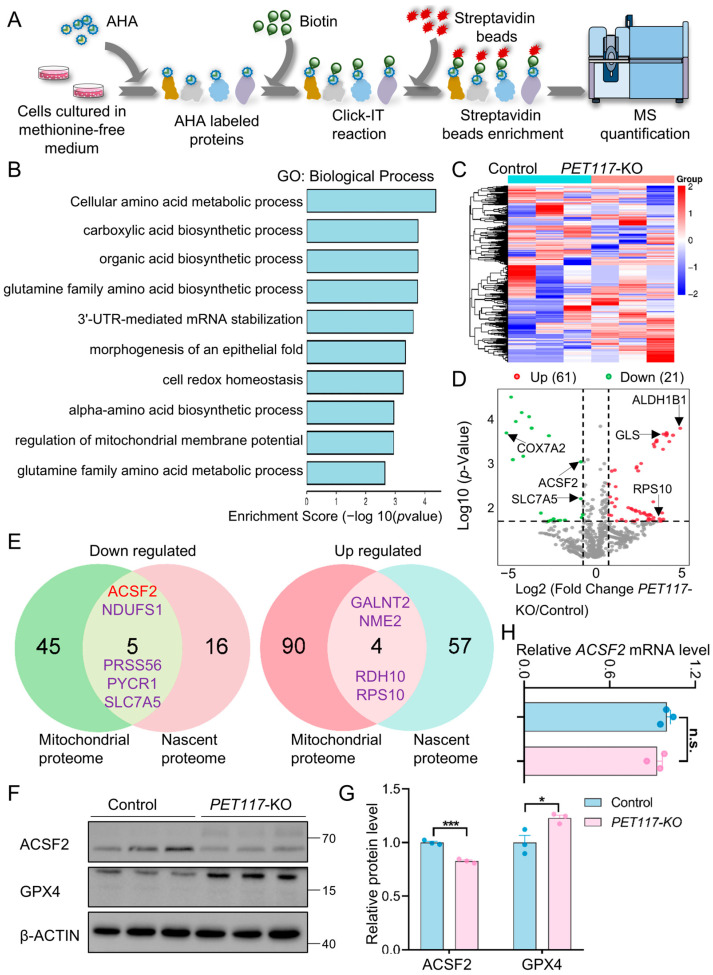
PET117 promotes ferroptosis partially through maintaining ACSF2 protein abundance. (**A**) Schematic diagram of AHA-labeled nascent proteome detection. (**B**) GO enrichment analysis of proteins with significant difference (*PET117*-KO versus control, cut-off at |log2-fold change| ≥ 1 and *p*-value < 0.05). (**C**) Heatmaps of hierarchical clustering in protein levels of the control and *PET117*-KO HeLa cells (*n* = 3 biologically independent samples). (**D**) Volcano map of the control and *PET117*-KO HeLa cells. Genes with *p*-value < 0.05 are present above the horizontal lines (gray). Proteins with significant fold-change are outside the gray vertical lines (log2-fold change ≥ 1, *p*-value < 0.05, red; log2 fold change ≤ −1, *p*-value < 0.05, green; *n* = 3 biologically independent samples). (**E**) Venn diagram showing the number of significantly changed proteins identified in nascent proteome and mitochondrial proteome. (**F**,**G**) Immunoblotting assays of ACSF2 and GPX4 in the control and *PET117*-KO HeLa cells. β-ACTIN was used as loading controls. Quantified intensities were presented in bar graph (**G**) as means ± s.e.m. and analyzed by a two-tailed paired Student’s *t*-test (*n* = 3, *: *p* < 0.05, ***: *p* < 0.001). (**H**) qRT-PCR assays of *ACSF2* mRNAs in control and *PET117*-KO HeLa cells (*n* = 3, n.s., not significant). GO: Gene Ontology.

To identify the potential downstream targets of PET117, we performed bio-orthogonal labeling of nascent proteins in *PET117*-KO HeLa cells using the methionine analog azidohomoalanine (AHA) [29]. Following the pulse-labeling period, AHA-incorporated peptides were biotinylated via click chemistry and enriched using streptavidin beads for subsequent quantitative mass spectrometry analysis (Figure 6A, Supplementary Table S3). Gene Ontology (GO) analysis of the nascent proteome highlighted significant alterations in several biological processes (Figure 6B). The significant changes in nascent proteins were shown in the heatmap and volcano plot (Figure 6C,D).

By comparing the mitochondrial proteome with the nascent proteome, it was found that the altered proteins were significantly different (Figure 6E). Remarkably, ACSF2 emerged as a common downregulated protein in both datasets (Figure 6E). This finding was of particular interest because ACSF2 has recently been implicated in ferroptosis regulation [30,31]. We therefore investigated the mechanism underlying ACSF2 downregulation in *PET117*-deficient cells. Although *PET117* knockout markedly reduced ACSF2 protein levels (Figure 6F,G), *ACSF2* mRNA levels remained unchanged as determined by qPCR (Figure 6H), suggesting that PET117 regulates ACSF2 at the post-transcriptional or translational level. In addition, GPX4 protein expression was significantly elevated in *PET117*-KO cells (Figure 6F), consistent with the observed ferroptosis-resistant phenotype. Although ACSF2 protein abundance was consistently reduced in PET117-deficient cells, the present study demonstrates an association rather than a direct causal relationship between ACSF2 downregulation and ferroptosis resistance. Therefore, ACSF2 should currently be considered a potential downstream mediator associated with PET117 deficiency. Future rescue experiments restoring ACSF2 abundance will be required to determine whether ACSF2 directly mediates the effects of PET117 on ferroptosis.

### 3.7. ACSF2 Is Positively Associated with PET117 and Ferroptosis-Related Genes in Cervical Cancer

**Figure 7:**
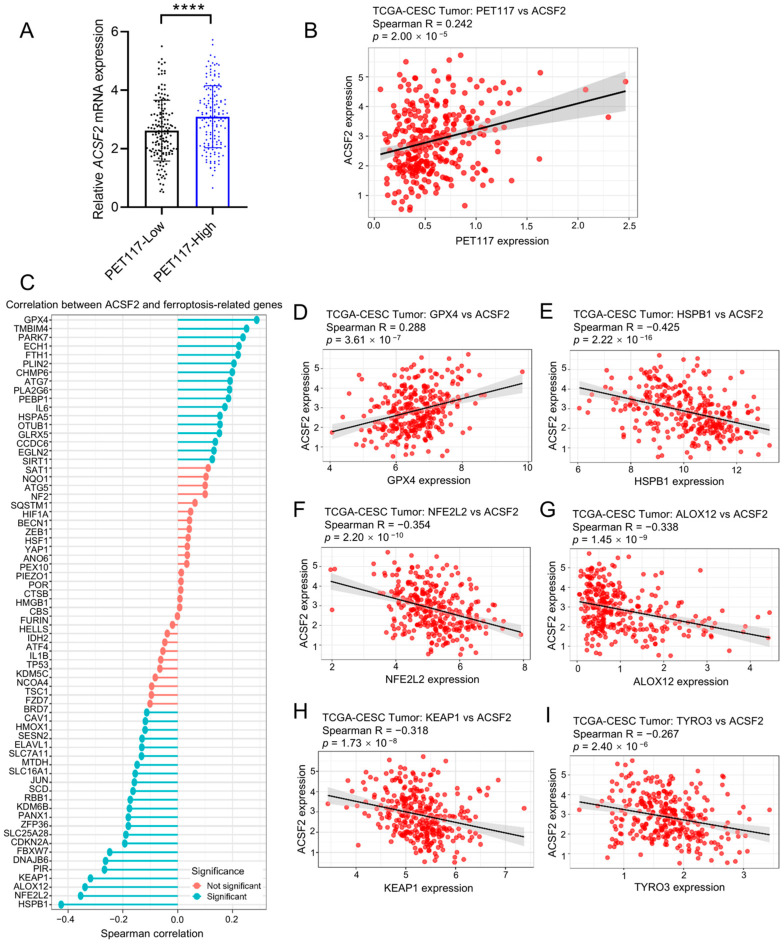
*ACSF2* is positively associated with *PET117* and ferroptosis-related genes in cervical cancer. (**A**) Comparison of *ACSF2* mRNA expression between *PET117*-high and *PET117*-low groups in TCGA-CESC tumor samples. Samples were stratified according to the median expression level of *PET117*. Statistical significance was determined using a Student’s *t*-test assuming equal variances. **** *p* < 0.0001. (**B**) Scatter plot showing the positive correlation between *PET117* and *ACSF2* in TCGA-CESC tumors. Spearman correlation coefficient (R) and corresponding *p* value are indicated. (**C**) Spearman correlation analysis between *ACSF2* and ferroptosis-related genes in TCGA-CESC. Genes significantly correlated with *ACSF2* are highlighted in cyan, whereas non-significant genes are shown in salmon. Positive and negative correlations are ranked according to the correlation coefficient. (**D**–**I**) Representative scatter plots with the highest correlation coefficient showing correlations between ACSF2 and selected ferroptosis-related genes, including positively correlated genes. Spearman correlation coefficients and corresponding *p* values are indicated in each panel.

To validate the clinical relevance of the PET117-ACSF2 regulatory axis identified in our cellular models, we analyzed the relationship between PET117 and ACSF2 in the TCGA-CESC cohort. *ACSF2* mRNA expression was significantly elevated in the *PET117*-high group compared with the *PET117*-low group (Figure 7A). In agreement with this, Spearman correlation analysis demonstrated a significant positive correlation between PET117 and ACSF2 expression in cervical cancer tissues (Figure 7B).

We further analyzed the association between ACSF2 and ferroptosis-related genes. ACSF2 expression showed significant correlations with multiple ferroptosis-associated genes (Figure 7C). Specifically, ACSF2 positively correlated with GPX4, whereas it negatively correlated with HSPB1, NFE2L2, ALOX12, KEAP1 and TYRO3 (Figure 7D–I). These findings support a potential role for ACSF2 as a potential downstream mediator of PET117 in ferroptosis-associated metabolic regulation in cervical cancer.

## 4. Discussion

PET117 have been linked to mitochondrial disorders and oxidative phosphorylation defects, but its role in cancer biology remains largely unexplored. In the present study, bioinformatic analyses revealed that PET117 was significantly upregulated in cervical cancer tissues in both the GSE63514 and TCGA-CESC cohorts. Interestingly, despite its elevated expression in cervical cancer tissues, PET117 expression was not significantly associated with overall survival in the TCGA-CESC cohort. In this study, we identify PET117 as a novel regulator of ferroptosis in cervical cancer, consistent with a previous conference abstract reporting that PET117 promotes ferroptosis in colorectal cancer cells through regulation of mitochondrial metabolism [32]. Our data demonstrate that *PET117* deficiency confers robust resistance to erastin- and RSL3-induced ferroptosis by reducing iron accumulation and lipid peroxidation, indicating that PET117 positively regulates ferroptotic sensitivity. These findings suggest that PET117 may function primarily as a regulator of mitochondrial metabolism and ferroptosis-related processes rather than as an independent prognostic biomarker. Therefore, elucidating the mechanistic role of PET117 in ferroptosis may provide more biologically relevant insights than evaluating its prognostic significance alone.

Ferroptosis represents a form of regulated necrosis precipitated by the uncontrolled accumulation of lipid hydroperoxides [33,34]. Although the core machinery—comprising System Xc^−^, GPX4, and FSP1—has been extensively characterized, the precise involvement of mitochondrial components in modulating ferroptotic susceptibility remains contentious and under active investigation [35,36]. However, our results reveal a distinct function of PET117 in ferroptosis regulation. The ferroptosis-resistant phenotype of *PET117*-deficient cells was accompanied by reduced iron accumulation and an enhanced antioxidant state. Proteomic analyses revealed decreased FTH1 levels and remodeling of the mitochondrial thioredoxin system, including altered TXN2 and PRDX6 expression. In addition, upregulation of CHCHD2 and GFER suggests activation of mitochondrial protective responses that may preserve cristae integrity and redox homeostasis [37,38]. Collectively, these alterations likely buffer oxidative stress and thereby suppress the execution of ferroptosis.

Notably, we also observed increased GPX4 expression in *PET117*-KO cells. GPX4 is widely recognized as a central negative regulator of ferroptosis because it detoxifies phospholipid hydroperoxides and prevents the accumulation of lethal lipid peroxidation products [39]. Previous studies have demonstrated that GPX4 inhibition is sufficient to trigger ferroptosis, whereas elevated GPX4 expression promotes resistance to ferroptotic cell death [40]. Therefore, the increased GPX4 expression observed following *PET117* depletion may represent an adaptive antioxidant response that contributes to the ferroptosis-resistant phenotype. This interpretation is consistent with previous studies showing that maintenance of cellular redox homeostasis is a critical determinant of cell fate. For example, Zhai et al. reported that melatonin suppresses oxidative stress through the MAP3K8/FOS pathway to protect granulosa cells from apoptosis, highlighting the importance of antioxidant defense mechanisms in regulating programmed cell death [41]. Their study focused on apoptosis rather than ferroptosis, it further supports the concept that modulation of oxidative stress is a common mechanism underlying multiple forms of regulated cell death. Together with the reduced ROS levels and altered mitochondrial function detected in *PET117*-KO cells, GPX4 upregulation may provide an additional mechanism by which PET117 deficiency suppresses ferroptosis. Although the precise molecular connection between PET117 and GPX4 remains unclear, our findings suggest that GPX4 may function as an important downstream effector associated with ferroptosis resistance in PET117-deficient cells. This observation further supports the concept that mitochondrial dysfunction and antioxidant defense systems cooperatively regulate ferroptosis susceptibility.

ACSF2 participates in fatty acid activation and lipid remodeling, ACSF2 downregulation may reduce the generation of oxidizable polyunsaturated fatty acid substrates and ultimately decrease lipid peroxidation susceptibility. Bioinformatic studies have identified ACSF2 in ferroptosis-related prognostic signatures in acute myeloid leukemia [30], and functional experiments confirmed that ACSF2 knockdown suppresses ferroptosis [31]. Shi et al. reported that ACSF2 is a key factor in ferroptosis regulation in ulcerative colitis [42]. Notably, our previous study revealed that PET117 directly interacts with and stabilizes the mitochondrial translation activator TACO1, thereby promoting the synthesis of mitochondria-encoded COX1, a core catalytic subunit of complex IV. Loss of PET117 reduces TACO1 stability, suppresses COX1 translation, decreases cytochrome c oxidase activity, and impairs mitochondrial oxygen consumption and ATP production [23]. Based on this, we propose a working model in which PET117 functions upstream of ACSF2 through regulation of mitochondrial respiratory chain integrity. Specifically, PET117 deficiency may impair complex IV assembly via destabilization of TACO1 and suppression of COX1 translation, resulting in OXPHOS dysfunction, ACSF2 downregulation, and ferroptosis resistance. Further studies are required to determine whether ACSF2 is regulated through mitochondrial retrograde signaling pathways downstream of PET117-mediated respiratory dysfunction.

The identification of ACSF2 as a potential downstream effector provides a mechanistic link between PET117 and ferroptosis regulation. Interestingly, PET117 deficiency significantly reduced ACSF2 protein abundance without significantly affecting ACSF2 mRNA expression, suggesting a post-transcriptional regulatory mechanism. PET117 is a mitochondrial cytochrome c oxidase assembly factor required for proper complex IV biogenesis and mitochondrial homeostasis [17]. Previous studies have shown that disruption of PET117 impairs mitochondrial respiratory chain function and alters mitochondrial protein homeostasis [43]. Given that ACSF2 is a nucleus-encoded mitochondrial acyl-CoA synthetase involved in fatty acid activation and lipid metabolism, PET117 deficiency may compromise mitochondrial protein import, maturation, or folding processes, thereby decreasing ACSF2 protein stability. Alternatively, mitochondrial stress induced by PET117 loss may activate mitochondrial protein quality-control pathways, resulting in accelerated degradation of ACSF2 through mitochondrial proteases or the ubiquitin–proteasome system. Although these mechanisms remain to be experimentally validated, our findings suggest that PET117 maintains ACSF2 abundance primarily at the protein level rather than through transcriptional regulation.

Several limitations of this study should be acknowledged. First, our mechanistic investigations were conducted primarily in HeLa cells, and therefore the generalizability of the observed effects of PET117 on ferroptosis regulation across different cervical cancer models remains to be determined. Validation in additional cervical cancer cell lines with distinct genetic backgrounds, such as SiHa, CaSki, and C33A, will be necessary to determine whether the PET117–ACSF2 axis is broadly applicable in cervical cancer. Second, although our results demonstrated that PET117 deficiency decreased ACSF2 abundance, the mechanistic link between PET117 and ACSF2 remains only partially demonstrated. Future studies employing PET117 overexpression, ACSF2 re-expression (rescue), and ACSF2 knockdown approaches will be necessary to determine the precise contribution of the PET117–ACSF2 axis to ferroptosis regulation. Third, although our results demonstrate that PET117 deficiency suppresses ferroptosis sensitivity through ACSF2 downregulation, we did not comprehensively characterize ferroptosis-associated mitochondrial and lipid metabolic alterations. Canonical ferroptosis assessments such as C11-BODIPY-based lipid ROS detection, GPX4 enzymatic activity measurements, ultrastructural examination of mitochondria by transmission electron microscopy, and ferroptosis-related lipidomic analyses were not performed. Given the established roles of mitochondrial dysfunction and lipid peroxidation in ferroptosis, future studies incorporating these approaches will be important for elucidating the detailed molecular events downstream of the *PET117*–ACSF2 axis and for defining the contribution of mitochondrial lipid metabolism to ferroptosis regulation in cervical cancer. Fourth, no in vivo validation was included in the current study, which restricts the evaluation of the biological significance of *PET117*-mediated ferroptosis regulation in a physiological context. Future studies involving multiple cervical cancer models and animal experiments will be required to further validate our findings. Therefore, while our results reveal a previously unrecognized role of PET117 in ferroptosis regulation, the potential clinical or therapeutic implications of these findings should be interpreted with caution until further evidence becomes available.

In conclusion, we uncover a previously unrecognized function of the mitochondrial protein PET117 in promoting ferroptosis in cervical cancer. Mechanistically, PET117 promotes ferroptosis at least partly through maintaining ACSF2 protein abundance. These findings expand the repertoire of mitochondrial proteins involved in cell death regulation and suggest that the PET117-ACSF2 axis may represent a potential therapeutic target for cervical cancer.

## Data Availability

The experimental or analytical data from this study are available upon request from the corresponding author. The RNA-seq data generated in this study have been deposited in the NCBI BioProject database under accession number PRJNA1478756 (https://www.ncbi.nlm.nih.gov/bioproject/PRJNA1478756, accessed on 7 July 2026). The corresponding BioSample accessions are SAMN60909388–SAMN60909393 (https://www.ncbi.nlm.nih.gov/biosample/SAMN60909388, https://www.ncbi.nlm.nih.gov/biosample/SAMN60909389, https://www.ncbi.nlm.nih.gov/biosample/SAMN60909390, https://www.ncbi.nlm.nih.gov/biosample/SAMN60909391, https://www.ncbi.nlm.nih.gov/biosample/SAMN60909392, https://www.ncbi.nlm.nih.gov/biosample/SAMN60909393, accessed on 7 July 2026). The associated SRA records have been submitted to NCBI (Submission ID: SUB16258325) (https://www.ncbi.nlm.nih.gov/sra/?term=PRJNA1478756, accessed on 7 July 2026). Additional data supporting the findings of this study are available from the corresponding author upon reasonable request.

## Supplementary Materials

The following supporting information can be downloaded at: https://www.mdpi.com/article/10.3390/antiox15070876/s1, Figure S1: Clinical relevance of PET117 expression in cervical cancer based on TCGA-CESC data. Figure S2: Immunoblotting assays of PET117 in the control and *PET117*-KO HeLa cells. β-ACTIN was used as loading control; Table S1. Mitochondrial proteomics analysis of *PET117*-KO HeLa cells; Table S2. RNA sequencing (RNA-seq) analysis of *PET117*-KO HeLa cells; Table S3. AHA labeling nascent proteomic in *PET117*-KO HeLa cells; Table S4. qPCR primers used in this study.
