# Supplementary material for: PET117 Deficiency Confers Ferroptosis Resistance Through ACSF2 Downregulation in Cervical Cancer
Source: Antioxidants (Basel). 2026 Jul 14;15(7):876. doi: 10.3390/antiox15070876 (PMC13405933; doi:10.3390/antiox15070876)
Supplement: Supplementary file 1 [file antioxidants-15-00876-s001.zip › Supplementary Table S4.pdf]

Supplementary Table S4. qPCR primers used in this study.

Gene	Primers
STA1	F: ACCCGTGGATTGGCAAGTTAT; R: TGCAACCTGGCTTAGATTCTTC
ALOX12	F: ATGGCCCTCAAACGTGTTTAC; R: GCACTGGCGAACCTTCTCA
HSPA5	F: CATCACGCCGTCCTATGTCG; R: CGTCAAAGACCGTGTTCTCG
SLC7A11	F: GGTCCATTACCAGCTTTTGTACG; R: AATGTAGCGTCCAAATGCCAG
HMGB1	F: TATGGCAAAAGCGGACAAGG; R: CTTCGCAACATCACCAATGGA
HMOX1	F: AAGACTGCGTTCCTGCTCAAC; R: AAAGCCCTACAGCAACTGTCG
NQO1	F: GAAGAGCACTGATCGTACTGGC; R: GGATACTGAAAGTTCGCAGGG
COX14	F: TGCAGTGTCCGAGTCTACCA; R: AGGTCTTCTGTTCTTCTGCGG
ATF4	F: CCCTTCACCTTCTTACAACCTC; R: TGCCCAGCTCTAAACTAAAGGA
ANO6	F: AAATTGCCTCTGAAACCCAATGA; R: GCTTTCGTCTACACTGAGGACTT
NCOA4	F: GCTCAGCAGCTCTACTCGTTA; R: GGCACACAGAGACTTGATTGG
PANX1	F: TTTACAACCGTGCAATTAAGGCT; R: AAGTTCTCGGTAACACCTGGA
CBS	F: GGCCAAGTGTGAGTTCTTCAA; R: GGCTCGATAATCGTGTCCCC
ATG7	F: ATGATCCCTGTAACTTAGCCCA; R: CACGGAAGCAAACAACTTCAAC
ACSF2	F: CCAGGCTATGGAACTGGAGTA; R: CTGGGTCTTGAATTGCTTGGG
GAPDH	F: CAGCAAGAGCACAAGAGGAA; R: AGGGGTCTACATGGCAACTG
