# Supplementary material for: PET117 Deficiency Confers Ferroptosis Resistance Through ACSF2 Downregulation in Cervical Cancer
Source: Antioxidants (Basel). 2026 Jul 14;15(7):876. doi: 10.3390/antiox15070876 (PMC13405933; doi:10.3390/antiox15070876)
Supplement: Supplementary file 1 [file antioxidants-15-00876-s001.zip › Supplementary Figures S1 and S2.pdf]

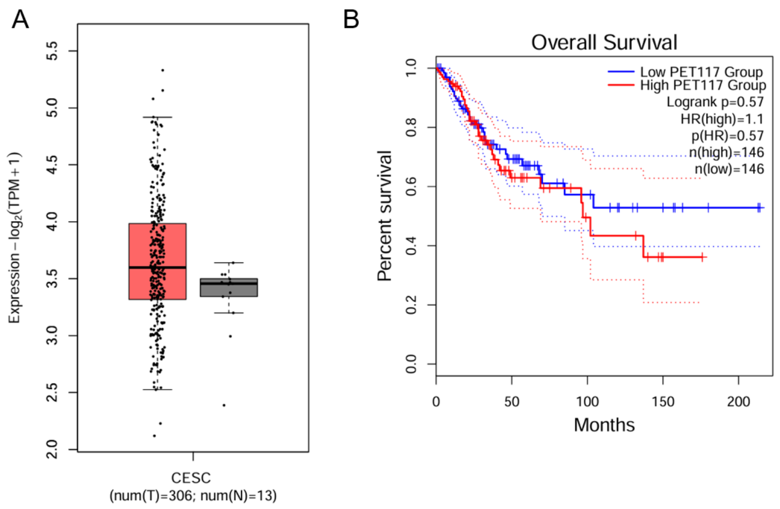

Supplementary Figure S1. Clinical relevance of PET117 expression in cervical cancer based on TCGA-CESC data. (A) Differential expression analysis of PET117 between cervical cancer tissues and normal cervical tissues using the GEPIA2 platform. (B) Kaplan–Meier overall survival analysis of cervical cancer patients stratified by PET117 expression using the GEPIA2 platform. Patients were divided into high- and low-expression groups according to the median PET117 expression level.

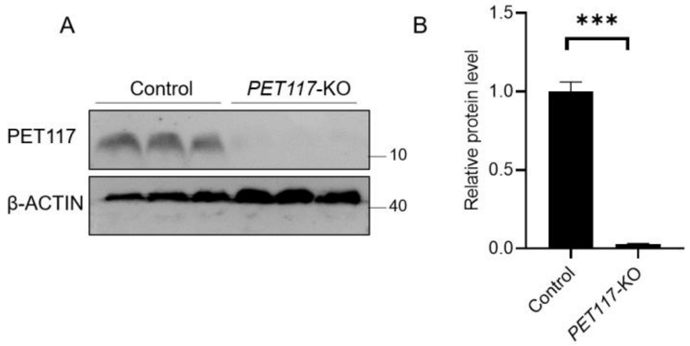

Supplementary Figure S2. (A) Immunoblotting assays of PET117 in the control and PET117-KO HeLa cells. β-ACTIN was used as loading control. (B) Quantified intensities were presented in bar graph as means ± s.e.m. and analyzed by a two-tailed paired Student’s t-test (n = 3, ***: P < 0.01).
